# Performance of the Parasympathetic Tone Activity (PTA) index to assess the intraoperative nociception using different premedication drugs in anaesthetised dogs

**DOI:** 10.1080/23144599.2020.1783090

**Published:** 2020-07-07

**Authors:** Christelle Mansour, Nour El Hachem, Patrick Jamous, Georges Saade, Emmanuel Boselli, Bernard Allaouchiche, Jeanne-Marie Bonnet, Stéphane Junot, Rana Chaaya

**Affiliations:** a VetAgro Sup, APCSE unit, Campus Vétérinaire De Lyon, Université De Lyon, Marcy-L’étoile, France; b Lebanese Faculty of Agronomy and Veterinary Medicine, Beirut, Lebanon; c Oniris, École Nationale Vétérinaire Agroalimentaire et de l’Alimentation, Nantes, France

**Keywords:** PTA, haemodynamic reactions, premedication, monitoring, dog

## Abstract

The dynamic variations of the Parasympathetic Tone Activity (PTA) index were evaluated to assess nociception in dogs undergoing anaesthetic protocols with different premedication drugs. Sixty-six dogs, divided into three groups of 22 dogs each, were given different premedication drugs “morphine” (0.2 mg/kg), “morphine + medetomidine (5 μg/kg)”, “morphine + acepromazine (0.03 mg/kg)”, then similarly induced and maintained under general anaesthesia. The PTA, HR (heart rate) and MAP (mean arterial pressure) were assessed at S (steady-state), Cut (cutaneous incision), PTA_E_ (PTA Event, assessed 1 min before a significant PTA decrease), HDR (Haemodynamic reaction, assessed 5 min before an increase of ≥ 20% in HR and/or MAP). For each group, the dynamic variation of PTA (ΔPTA) was calculated and a Receiver Operating Characteristics (ROC) curve was performed to detect if any of the premedication drugs may alter the performance of PTA index to predict intraoperative haemodynamic reactions. In all groups, a haemodynamic reaction was detected after Cut, PTA_E_ and HDR and was preceded with a significant decrease of PTA, except for “morphine + medetomidine” group which showed a significant drop of PTA only at PTA_E_ and HDR. The ΔPTA showed a fair performance in all groups (a mean [95% CI] AUC of 0.73 [0.62–0.82], 0.70 [0.59–0.79] and 0.71 [0.59–0.80] respectively in morphine, morphine + ACP and morphine + medetomidine). Although ΔPTA was rather altered by the premedication drug, it was able to predict, to a certain extent, haemodynamic reactions in all groups.

## 1. Introduction

In anaesthesia, the vital parameters such as the heart rate, respiratory rate and arterial pressure are monitored to evaluate changes of the autonomic nervous system (ANS) [1]. However, these parameters are not specific and may be influenced by anaesthetic agents and surgical procedures [2]. Thus, it is interesting to measure directly the changes in ANS to predict the effects of anaesthetic drugs on the vital parameters [3].

In human and veterinary medicine, the heart rate variability (HRV) seems to be a sensitive and non-invasive method to assess ANS during general anaesthesia [4]. Therefore, changes in HRV that occur during surgical procedures and drug administration allow anaesthesiologists to better understand the impact of drugs administered on the regulation of the ANS [5].

Recently, a new index called Parasympathetic Tone Activity (PTA, MDoloris Medical Systems, Lille, France) has been developed in veterinary medicine. It is similar to the Analgesia Nociception Index (ANI, MDoloris Medical Systems, Lille, France), used for the assessment of nociception in humans [6–8]. Both indexes are based on the HRV measurement to estimate the parasympathetic tone during nociceptive stimulations. They are scored between 0 and 100: High index values indicate prominent parasympathetic tone, as observed during adequate analgesia. In case of nociception, sympathetic tone increases and parasympathetic tone decreases, leading to decreased index values and haemodynamic reactivity. A clinical study showed that a dynamic variation of PTA (ΔPTA) of −18% can predict changes within 5 min in the vital parameters during general anaesthesia in dogs using morphine in premedication [9]. In addition, a recent experimental study comparing the PTA monitor with cardiovascular changes in healthy dogs revealed that PTA index could be more efficient in detecting low intensity nociceptive stimuli than those eliciting cardiovascular changes [10].

In anaesthesia, different protocols are used routinely in dogs including opioids, alpha2-adrenoceptor agonists, phenothiazine derivatives and others. These drugs are known to affect the vital parameters through their action on ANS. Therefore, our hypothesis was that the performance of PTA index to predict intraoperative haemodynamic reactions in dogs anaesthetised with different anaesthetic protocols could be altered due to the different intraoperative analgesic effect of the drugs.

## 2. Materials and methods

### 2.1. Ethical statement

This clinical trial received the institutional approval of the ethical committee of VetAgro Sup (n°1514) as well as the informed consent of the owners.

### 2.2. Animals

Six-six healthy dogs were prospectively enrolled in this study and were admitted for elective surgeries (abdominal, orthopaedic and cutaneous surgeries) under general anaesthesia. Based on the physical examination and, where required, laboratory tests, all dogs were of American Society of Anaesthesiologists (ASA) Physical Status I or II. Non-inclusion criteria included dogs with cardiac or autonomic disease, dogs with aggressive temperament, old dogs (> 7 yrs), those presenting an ASA more than II, and brachycephalic dogs. Exclusion criteria consisted of dogs requiring cardiopulmonary resuscitation or the administration of intraoperative continuous rate infusion.

### 2.3. Anaesthetic protocols

After 8 hours of fasting, the recruited animals were anaesthetised.

An hour prior to the administration of the premedication drug, all dogs received a non-steroidal anti-inflammatory drug, meloxicam (Meloxivet ^–^ Impact Bio Pharma – India) 0.2 mg/kg IV, as well as an antibiotic injection of cefazolin (Hikma Cefazolin – HIKMA pharmaceuticals – Jordan), 20 mg/kg. Based on the agent used in premedication, the animals were divided randomly into 3 groups: “morphine” group receiving only morphine 0.2 mg/kg IV (morphine chlorhydrate – Aguettant – France), “morphine + medetomidine” group receiving morphine 0.2 mg/kg IV followed, 5 min later, by an injection of medetomidine 5 μg/kg IV (Domitor ^–^ medetomidine hydrochloride – Pfizer Animal Health – USA) and “morphine + ACP” group receiving an IM injection of acepromazine (ACP) 0.03 mg/kg (Calmivet – Solution injectable – Vetoquinol – France) followed, 20 minutes later, by an injection of morphine 0.2 mg/kg IV. The interval of 20 minutes was employed due to the relatively slow onset of sedation following administration of acepromazine compared to medetomidine. Afterwards, the animals were administered diazepam 0.2 mg/kg IV (diazepam TVM – France) and 4 mg/kg of propofol IV (Propovet – Axience – France) given to effect. As the loss of reflexes was achieved, the anaesthesia was maintained by inhalation of 1.5% of isoflurane (Isoflo – Axience – France) in 100% oxygen after orotracheal intubation.

Intraoperatively, all groups received a rate of 5 mL/kg/h of intravenous fluids (Ringer lactate). Signs of anaesthetic depth were continuously assessed including absence of reflexes (palpebral reflex, corneal reflex, swallowing reflex), presence of lacrimation and absence of involuntary muscle movement. Rescue analgesia, consisting in a bolus of fentanyl 1 μg/kg was provided in case of haemodynamic response due to nociception. In orthopaedic surgeries, a bolus of morphine 0.1 mg/kg IV was given at one-hour interval to maintain a constant concentration in the bloodstream.

### 2.4. Anaesthesia monitoring and PTA measurement

Vital parameters, including Mean Arterial Pressure (MAP) (mmHg) and Heart Rate (HR) (bpm) were continuously monitored (Advisor® Vital Signs Monitor V9203®, Surgivet, USA) and systematically collected every 5 min during the procedures. Arterial pressure measurement was repeated twice at times of interest using an oscillometric non-invasive method. The depth of anaesthesia was assessed clinically and the concentration of inhalant anaesthetic was adjusted.

The PTA index was obtained from each dog using the PhysioDoloris® monitor (MDoloris Medical Systems – Lille – France) connected to the animal’s multiparameter monitor. This non-invasive device uses the ECG analogue output from the monitor and displays continuously an average measurement of PTA over the previous 4 min. Details on PTA calculation have been described elsewhere [9].

### 2.5. Study design

**Figure 1.**
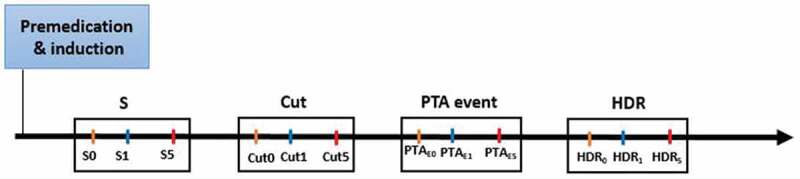
Surgical times of interest

PTA, HR and MAP as well as their variations (at 1 min and 5 min thereafter) were recorded at predefined time points (Figure 1): S (steady-state; before any surgical stimulus of the anaesthetised dogs), Cut (first cutaneous incision), PTA_E_ (PTA Event, a retrospective time defined as a significant PTA decrease within 1 min (PTA_E1_) in comparison with baseline value (PTA_E0_)), and HDR (Haemodynamic Reaction, a retrospective time defined as an increase of ≥ 20% in HR and/or MAP within 5 min (HDR_5_) in comparison with baseline value (HDR_0_)). In other words, PTA_E_ aimed to evaluate if a decrease of PTA was associated with a haemodynamic reaction 5 min thereafter, whereas HDR was designed to evaluate if a haemodynamic reaction was associated 5 minutes earlier with a drop of PTA. The interval of 5 min between the drop of PTA and the occurrence of a haemodynamic reaction was used owing to the results obtained in our previous study where a significant decrease of PTA was detected followed by a haemodynamic reaction within 5 min after the nociceptive stimuli [9].

Based on a recent report showing better performance of ΔPTA than static values of the index to predict haemodynamic response in anaesthetized dogs [9], ΔPTA was also calculated as followed:$\Delta PTA=[(PTA_{1min}-PTA_{init})/\left(PTA_{1min}+PTA_{init}\right)/2]*$

100, where PTA_init_ is the value of PTA at the predefined time and PTA_1_ _min_ is the value of PTA 1 min thereafter. The performance of ΔPTA to predict haemodynamic reactions in each group was therefore assessed using Receiver Operating Characteristics (ROC) curves analysis of the pooled data of ΔPTA.

### 2.6. Statistical analysis

Statistical analysis was performed using Med-Calc^@^ 12.1.4.0 (MedCalc Software® – Mariakerke – Belgium). Normality of distribution was assessed using the Kolmogorov-Smirnov test. Non-parametric data were expressed as median [IQR]. Friedman’s test was used to detect significant variations for MAP, HR and PTA between time-points within groups. Kruskal-Wallis test was used to compare MAP, HR and PTA variations between groups. For each group, ΔPTA calculated over 1 min at each time-point was used to perform a Receiver Operating Characteristics (ROC) curve to detect the performance of ∆PTA to predict an increase of MAP and/or HR within 5 min. A p-value < 0.05 was considered statistically significant.

## 3. Results

### 3.1. Animals

Sixty-six dogs of different breeds undergoing elective surgeries were enrolled in this study (29 males, 37 females; mean age 2.8 ± 3 yrs; mean body weight 19.7 ± 11.2 kg and ASA I or II) and divided equally into 3 groups: “morphine” (22 dogs), “morphine + medetomidine” (22 dogs) and “morphine + acepromazine” (22 dogs). No differences in sex, age and weight were detected between the groups (p > 0.07). The anaesthetic duration appeared to be longer in “morphine + medetomidine” group (142.3 ± 52.9 min) compared to “morphine” (91 ± 44.5 min) and “morphine + acepromazine” (62.9 ± 26.8 min) (p < 0.0001). As for the inhaled Isoflurane, no significant difference was found in the end tidal Isoflurane (Et_Iso_) measured throughout the surgeries in all groups (“morphine” 2.1 ± 0.4; “morphine + medetomidine” 1.9 ± 0.2 and “morphine + acepromazine” 2 ± 0.1, p = 0.2). Finally, no significant difference was found between groups with regard of the remedy for rescue analgesia (p > 0.5).

### 3.2. Evolution of PTA, MAP and HR at the surgical time-points

For each surgical time (S, Cut, PTA_E_ and HDR), the variation of PTA, HR and MAP was assessed in each group.

In morphine group (Table 1), at S no significant change was detected in any parameter. PTA appeared to decrease significantly at Cut, PTA_E_ and HDR (Cut: −23%, p < 0.0001; PTA_E_: −17%, p < 0.0001 and HDR: −20%, p = 0.0009). HR increased significantly at Cut and PTA_E_ (Cut: +3.5%, p = 0.02, PTA_E_: +11.6%, p = 0.02) whereas MAP increased at all surgical time-points (Cut: +28%, p = 0.0004, PTA_E_: +2.2%, p = 0.02 and HDR: +26.2%, p = 0.0001).

**Table 1.** Evolution of PTA, HR and MAP in morphine group at each time-point

	TIME-POINTS			
PARAMETERS	S	Cut	PTA_E_	HDR
**PTA** **HR** **MAP**	+2.9% -3% 0%	−23%* +3.5%* +28%*	−17%* +11.6%* +2.2%*	−20%* +2.7% +26%*

**S**, steady-state; **Cut**, cutaneous incision; **PTA_E_**, PTA Event, assessed 1 min before a decrease significant PTA; **HDR**, Haemodynamic Reaction, assessed 5 min before an increase of ≥ 20% in HR and/or MAP.

* Indicates a significant rate of change (p < 0.05) of PTA at 1 minute and of HR and MAP at 5 min at the time-point of interest.

In morphine + ACP group (Table 2), an increase in PTA at S was observed (+11.3%, p < 0.0005). At Cut, PTA_E_ and HDR, a significant decrease in PTA (Cut: −21%, p < 0.0001; PTA_E_: −30%, p < 0.0001 and HDR: −9.5%, p = 0.03) was followed by a haemodynamic reaction (Cut: HR +10.8%, p = 0.002 and MAP +27%, p = 0.0001; PTA_E_: HR +7.4%, p = 0.03 and MAP +17.3%, p < 0.0001; HDR: MAP +25%, p = 0.001).

**Table 2.** Evolution of PTA, HR and MAP in morphine + ACP group at each time-point

	TIME-POINTS			
PARAMETERS	S	Cut	PTA_E_	HDR
PTA	+11.3%*	−21%*	−30%*	−9.5%*
HR	+1.6%	+10.8%*	+7.4%*	−0.8%
MAP	−1.7%	+27%*	+17.3%	+25%*

**S**, steady-state; **Cut**, cutaneous incision; **PTA_E_**, PTA Event, assessed 1 min before a decrease significant PTA; **HDR**, Haemodynamic Reaction, assessed 5 min before an increase of ≥ 20% in HR and/or MAP.

* Indicates a significant rate of change (p < 0.05) of PTA at 1 minute and of HR and MAP at 5 min at the time-point of interest.

In morphine + medetomidine group (Table 3), MAP decreased at S (−6.2%, p = 0.02) whereas HR increased (+7%, p = 0.03) with no change in PTA. At Cut, the increase in MAP (+7.1%, p = 0.002) was not accompanied with any decrease in PTA. At PTA_E_ and HDR, a progressive increase in HR (PTA_E_: +6.7%, p > 0.05; HDR: +13.9%, p = 0.02) and MAP (PTA_E_: +18.9%, p > 0.05; HDR: +20%, p = 0.0001) was observed with a previous significant decrease in PTA (PTA_E: −2_5%, p < 0.0001; HDR: −14%, p = 0.006).

**Table 3.** Evolution of PTA, HR and MAP in morphine + medetomidine group at each time-point

	TIME-POINTS			
PARAMETERS	S	Cut	PTA_E_	HDR
PTA	+2%	0%	−25%*	−14%*
HR	+7%*	+8.5%	+6.7%	+13.9%*
MAP	−6.2%*	+7.1%*	+18.9%	+20%*

**S**, steady-state; **Cut**, cutaneous incision; **PTA_E_**, PTA Event, assessed 1 min before a decrease significant PTA; **HDR**, Haemodynamic Reaction, assessed 5 min before an increase of ≥ 20% in HR and/or MAP.

* Indicates a significant rate of change (p < 0.05) of PTA at 1 minute and of HR and MAP at 5 min at the time-point of interest.

### 3.3. Comparison of the variation of PTA, MAP and HR between groups at the surgical time-points

The comparison of the variation of PTA, HR and MAP between groups at each surgical time was also studied.

Regarding PTA variation, at Cut, a significant difference was shown between morphine + medetomidine and the 2 other groups. Morphine group and morphine + ACP group demonstrated a significant decrease in PTA (−23% and −21% respectively) compared to morphine + medetomidine which presented no variation (p < 0.0001). In addition, at PTA_E,_ PTA appeared to decrease more significantly in morphine + ACP group compared to morphine group (−30% *vs* −17% respectively) (p = 0.02).

In morphine + medetomidine group, the increase in MAP (+7.1%) appeared to be significantly less important than the 2 other groups (+28% and +27% respectively in morphine and morphine + ACP groups) (p = 0.0004). However, at PTA_E,_ the increase in MAP was significantly greater in morphine + medetomidine group (+18.9%) compared to morphine group (2.2%) (p = 0.05).

As for the variation of HR, no significant difference was found between groups at any surgical time-point.

### 3.4. Relationship between PTA and haemodynamic reactivity

**Figure 2.**
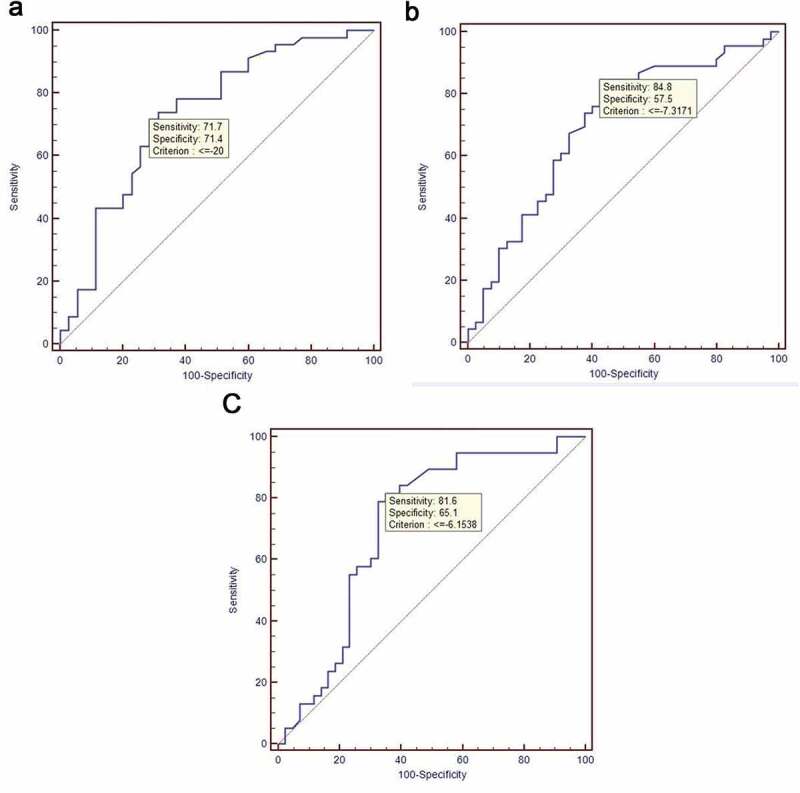
Performance of PTA to predict haemodynamic response in morphine (a), morphine + ACP (b) and (c) morphine + medetomidine groups

Morphine group (Figure 2(a)) showed a mean [95% CI] AUC of 0.73 [0.62–0.82], (p < 0.0001). A threshold value of −20% was found with a sensitivity and specificity of 71.7% and 71.4%, respectively. On the other hand, the mean [95% CI] AUC of morphine + ACP group (Figure 2(b)) was shown to be 0.70 [0.59–0.79], (p = 0.0005) accompanied with a threshold value of −7.3% and a sensitivity of 84.8% and specificity of 57.5%. Lastly, morphine + medetomidine group (Figure 2(c)) showed a mean [95% CI] AUC of 0.71 [0.59–0.80], (p = 0.0004), and a threshold value of −6.1% with a sensitivity of 81.6% and specificity of 65.1%. Therefore, low specificities were found for both morphine + ACP and morphine + medetomidine groups. In other words, in contrast to morphine group, ∆PTA appeared to identify less accurately intraoperative nociception in these two groups.

## 4. Discussion

The main purpose of this clinical study was to compare the effect of three anaesthetic protocols on the performance of PTA index. The PTA was assessed at nociceptive time-points (Cut, PTA_E_ and HDR) to investigate whether any predictive relationship exists between the parasympathetic tone and the haemodynamic reactions. This was performed in healthy animals using morphine alone or in combination with medetomidine and acepromazine.

Our results showed a haemodynamic reaction following a significant decrease in PTA during all nociceptive time-points (Cut, PTA_E_ and HDR) in all groups except at Cut in morphine + medetomidine group where an increase in MAP was noticed without any previous drop in PTA. Additionally, the performance of ΔPTA shown by the ROC curve analysis was found to be fair in predicting a haemodynamic reaction in each group. However, morphine + medetomidine and morphine + ACP groups revealed low specificities.

In the morphine group, the analysis of PTA in parallel with MAP and HR at different time-points showed that PTA decreased significantly at Cut, PTA_E_ and HDR and was followed by a significant haemodynamic response. These results coincide with a previous study showing that PTA index was associated with a correct performance (AUC ROC [95% CI] = 0.80 [0.71 to 0.88] (p < 0.05), with a sensitivity of 77% and a specificity of 72% for a threshold value of −18%) to assess nociception and predict a haemodynamic reactivity in anaesthetised dogs [9,11].

In morphine + ACP group, the haemodynamic reaction detected at all times-points was preceded with a decrease of PTA in a similar way as morphine group. These results were unexpected as the combination of ACP, a well-known sedative and anxiolytic drug, with an opioid should be advantageous thanks to the induced neuroleptanalgesia [12]. In addition, an intraoperative increase in MAP was unpredicted as the mechanism of action of acepromazine consists of blunting increase in blood pressure due to blockade of vascular alpha-1 adrenergic receptors causing a reduction in systemic vascular resistance and a consequent fall in blood pressure [13,14]. Therefore, in this study, one can assume that the addition of ACP couldn’t demonstrate any additional value compared to the administration of morphine alone. These findings are comprehensible as ACP itself is devoid from analgesic properties [15]. Thus, additional analgesic techniques should be considered before or during the operation such as, respectively, regional blocks and continuous rate infusion of an opioid, to avoid nociception and haemodynamic reactions.

Regarding morphine + medetomidine group, a decrease in MAP and increase in HR were noticed at steady-state with no apparent previous decrease in PTA. These haemodynamic changes could be related to the selectivity of medetomidine for central and peripheral alpha-2 receptors aiding in the modulation of stress response [16]. Another explanation for the decrease in MAP and increase in HR would be the reduction of the vasoconstrictive and bradycardic effects on medetomidine over time [16]. At Cut, morphine + medetomidine group showed a slight increase in MAP with no change in PTA. This is assumed to be related to the start of the surgery, however, the overwhelming central action of medetomidine at this surgical stage could have masked a detectable nociception represented by the lack of PTA decrease. During PTA_E_ and HDR, PTA decreased significantly in parallel to a perpetual increase of MAP and HR. A potential explanation could be that, at PTA_E_, the effect of medetomidine was waned and disappeared totally at HDR. We assume that this could be the result of the biotransformation of medetomidine over time reflecting a sympathetic activation and a decrease of the analgesic potency of medetomidine. In fact, medetomidine, which is an equal mixture of dexmedetomidine (responsible of the hypnotic/analgesic actions) and levomedetomidine (considered to be pharmacologically inactive) is quickly absorbed with peak plasma levels occurring in approximately 30 minutes and is rapidly eliminated from plasma [17,18]. Experimental and clinical evidence demonstrates that analgesia is not present throughout the entire period of sedation with alpha2-agonists and only lasts for half the duration of sedation [19]. Thus, the use of loco-regional anaesthesia [20] or continuous rate infusion of medetomidine during the operation might be needed to prolong the analgesic effect. When considering the ROC analysis of pooled data of ∆PTA at the predefined times, all groups showed a fair performance of ΔPTA to predict intraoperative haemodynamic reactivity. However, the specificity in the morphine + medetomidine and morphine + ACP groups appeared to be low. In addition, the threshold value of ΔPTA in morphine group (ΔPTA < −20%) was found to be more perceptible than in the morphine + medetomidine and morphine + ACP groups (ΔPTA < −6.1% and −7.3% respectively). The results in morphine group are compatible with those obtained in our previous study on ΔPTA performance to predict haemodynamic reaction in dogs anaesthetised using the same protocol [9]. In the morphine + medetomidine and morphine + ACP group, the ROC analysis could be explained by the effect of these drugs on the parasympathetic reflex loop, lowering the sympathetic activity and decreasing PTA response.

This study discloses several limitations. The number of dogs included in each group was limited which could have lowered the precision of ROC calculations. The dogs differed in size, age and breed and had potentially different autonomic nervous system activities. Moreover, the recruitment included dogs undergoing different types of surgeries with potentially different levels of pain. Nevertheless, the predefined times selected were independent of the type of surgery. However, this was unlikely to influence our results since the studied surgical time-points were designed to be independent of the procedures. The use of general anaesthesia as well as opioids in premedication and during the operation should also be considered a limitation since it could have influenced the analgesic and sedative status of the animals, thus impacting the ANS and consequently the PTA. Furthermore, the lack of enough prior research studies on the PTA index in animals compared to ANI in humans should also be considered a limitation.

## 5. Conclusion

Using different premedication drugs, the monitoring of the PTA index and its ΔPTA was associated with a fair performance to evaluate the analgesia nociception balance in anaesthetised dogs. Accordingly, it can be used in practice to facilitate the detection of intraoperative nociception in dogs. Our perspective would be to assess the plausible use of PTA index in managing drug administration intraoperatively (especially of opioids) with the intention of reducing the side effects of anaesthetic drugs.

**S**, steady-state, before any surgical stimulus of the anaesthetised dogs; **Cut**, first cutaneous incision; **PTA_E_**, PTA Event, retrospective time defined as a significant PTA decrease within 1 min (PTA_E1_) in comparison with baseline (PTA_E0_), and **HDR** (Haemodynamic Reaction, retrospective time defined as an increase of ≥ 20% in HR and/or MAP within 5 min (HDR_5_) compared to baseline (HDR_0_)).

(a) Area under the curve (AUC) = 0.73 [0.62–0.82]; (b) Area under the curve (AUC) = 0.70 [0.59–0.79]; (c) Area under the curve (AUC) = 0.71 [0.59–0.80].
